# Nitrogen dioxide component of air pollution increases pulmonary congestion assessed by lung ultrasound in patients with chronic coronary syndromes

**DOI:** 10.1007/s11356-021-17941-1

**Published:** 2021-12-09

**Authors:** Quirino Ciampi, Antonello Russo, Caterina D’Alise, Anna Ballirano, Bruno Villari, Cristina Mangia, Eugenio Picano, the Stress Echo 2030 study group of the Italian Society of Echocardiography, Cardiovascular Imaging

**Affiliations:** 1 Cardiology Division, Fatebenefratelli Hospital, Benevento, Italy; 2 Association for Public Health “Salute Pubblica”, Brindisi, Italy; 3 ARPA Campania, Naples, Italy; 4 CNR, ISAC- Institute of Sciences of Atmosphere and Climate, Lecce, Italy; 5 Biomedicine Department, CNR, Institute of Clinical Physiology, Pisa, Italy

**Keywords:** Air pollution, Coronary artery disease, Heart failure, Stress echocardiography

## Abstract

Pulmonary congestion is an intermediate biomarker and long-term predictor of acute decompensated heart failure.

To evaluate the effects of air pollution on pulmonary congestion assessed by lung ultrasound.

In a single-center, prospective, observational study design, we enrolled 1292 consecutive patients with chronic coronary syndromes referred for clinically indicated ABCDE-SE, with dipyridamole (*n* = 1207), dobutamine (*n* = 84), or treadmill exercise (*n* = 1). Pulmonary congestion was evaluated with lung ultrasound and a 4-site simplified scan. Same day values of 4 pollutants were obtained on the morning of testing (average of 6 h) from publicly available data sets of the regional authority of environmental protection. Assessment of air pollution included fine (< 2.5 µm diameter) and coarse (< 10 µm) particulate matter (PM), ozone and nitrogen dioxide (NO_2_).

NO_2_ concentration was weakly correlated with rest (*r* = .089; *p* = 0.001) and peak stress B-lines (*r* = .099; *p* < 0.001). A multivariable logistic regression analysis, NO_2_ values above the median (23.1 µg/m^3^) independently predicted stress B-lines with odds ratio = 1.480 (95% CI 1.118–1.958) together with age, hypertension, diabetes, and reduced (< 50%) ejection fraction. PM_2.5_ values were higher in 249 patients with compared to those without B-lines (median and IQR, 22.0 [9.1–23.5] vs 17.6 [8.6–22.2] µg/m^3^, *p* < 0.001). No other pollutant correlated with other (A-C-D-E) SE steps.

Higher concentration of NO_2_ is associated with more pulmonary congestion mirrored by B-lines at lung ultrasound. Local inflammation mediated by NO_2_ well within legally allowed limits may increase the permeability of the alveolar-capillary barrier and therefore pulmonary congestion in susceptible subjects.

ClinicalTrials.gov Identifier: NCT030.49995.

Heart failure is a major health problem and accounts for 5% of all hospital discharge diagnoses. Pulmonary congestion is the pathophysiological and clinical hallmark of heart failure and markedly increases days or weeks before episodes of acute decompensated heart failure (Bozkurt et al., 2021). Air pollution is a chronic, major risk factor for several cardiovascular diseases and accounts for over 20% of all cardiovascular deaths on a global scale. In 2019, air pollution was recognized as the fourth highest-ranking risk factor for mortality, with more attributable deaths than high LDL-cholesterol, high body mass index, physical inactivity, or tobacco use (Pinto et al., 2021). The acute worsening of air quality is a trigger of acute decompensated heart failure in susceptible patients at higher risk of cardiovascular events (Rajagopalan S, 2018). Several particulate and gaseous components of air quality have a damaging effect, well documented especially for fine particulate matter (PM_2.5_), ozone and nitrogen dioxide (NO_2_) (Newby DE, 2015). They may increase the permeability of the alveolar-capillary barrier and therefore pulmonary congestion in susceptible patients through complex molecular and cellular mechanisms characterized by increased inflammatory and oxyradical stress potentially impairing endothelial, smooth muscle cell, myocardial, alveolar, and neuronal function. The identification of triggers of acute cardiac decompensation in susceptible individuals is a major public health concern (Münzel T, 2021).

In the cascade of events leading to life-threatening acute decompensated heart failure, pulmonary congestion can be detected at a preclinical, asymptomatic stage by lung ultrasound as an accumulation of B-lines (also known as ultrasound lung comets) at rest and during stress. In patients with chronic coronary syndromes, B-lines are detectable in about 15% at rest and in an additional 15% only during stress. B-lines are a quantitative, direct sign of extravascular lung water accumulation, an intermediate biomarker of heart failure, and a long-term predictor of cardiovascular death (Scali MC, 2020).

The current study hypothesis was that ambient air quality and particularly same-day concentrations of NO_2_ and PM_2.5_ may affect pulmonary congestion detectable as rest and stress B-lines.

## Methods

### Study population

In this prospective study, we initially screened 1,340 patients referred from July 2016 to November 2020 to our hospital. Of these initial 1,340, 38 did not complete the full ABCDE stress echo (SE) protocol for missing information on step D (*n* = 38). An additional 10 patients were studied in days with no availability of same-day air quality data for logistic or technical reasons. The final study population included 1292 patients all studied with ABCDE protocol with diagnostic information available for all steps and same-day air quality data.

The inclusion criteria were the following: (1) age > 18 years; (2) referral for known or suspected chronic coronary artery disease (including dyspnea as the presenting symptom); (3) no severe primary valvular or congenital heart disease, or presence of prognosis-limiting comorbidities, such as advanced cancer, reducing life expectancy to < 1 year; (4) transthoracic echocardiography (TTE) of acceptable quality at rest and during stress; (5) willingness to give their written informed consent allowing scientific utilization of observational data, respectful of privacy rights; (6) availability of same-day air quality data.

All patients underwent resting TTE, lung ultrasound, and SE testing as part of a clinically-driven evaluation and according to the referring physician’s indications.

Written informed consent was obtained from all patients before testing. The study protocol was reviewed and approved by the institutional ethics committees as a part of the SE 2020–2030 study (148 – Comitato Etico Lazio-1, July 16, 2016; 148 – 291/294/295, March 8, 2021, Clinical trials. Gov Identifier NCT 030.49995). The study was funded partly by the Italian National Research Council (Ageing project, Progetto P001328, Progetto di Interesse-Invecchiamento) and with travel grants of the Italian Society of Echocardiography and Cardiovascular Imaging with dedicated sessions during national meetings. No support from the industry was received.

### Resting TTE and SE

We used commercially available ultrasound machines. All patients underwent comprehensive TTE at rest (Lang RM, 2015) and SE. Stress modalities were high dose dipyridamole (0.86 mg/kg over 6 min) in 1207 patients, high dose dobutamine in 84, and treadmill exercise in 1, with the protocols recommended by the European Association of Cardiovascular Imaging (Sicari R, 2009) and American Society of Echocardiography (Pellikka PA, 2020). Criteria for terminating the test were severe chest pain, diagnostic ST-segment shift, symptomatic hypotension, excessive blood pressure increase (systolic blood pressure ≥ 240 mmHg, diastolic blood pressure ≥ 120 mmHg), limiting dyspnea, maximal predicted heart rate, significant arrhythmias. Echocardiographic imaging was performed from parasternal long- and short-axis views, and apical 4- and 2-chamber views, using conventional 2-dimensional echocardiography. Anti-anginal drugs were usually not suspended before testing. Step A included an assessment of wall motion abnormalities. Wall motion score index (WMSI) was calculated at baseline and peak stress, in a four-point score ranging from 1 (normal) to 4 (dyskinetic) in a 17-segment model of the left ventricle. Step B of the protocol included the assessment of B-lines with lung ultrasound and the 4-site simplified scan, from mid-axillary to mid-clavicular lines on the third intercostal space, each site scored from 0 (normal horizontal A-lines) to 10 (white lung with coalescent B-lines) (Ciampi Q, 2021). Step C of the protocol included the force-based assessment of LVCR as the stress/rest ratio of force, calculated as systolic blood pressure/end-systolic volume (Ciampi Q, 2021). Coronary flow velocity reserve (step D) was assessed during the standard SE examination using intermittent imaging of wall motion and the left anterior descending coronary artery (Ciampi Q, 2021). Coronary flow in the mid-distal portion of the left anterior descending coronary artery was imaged from the low parasternal long-axis view and/or modified apical 2-, 3-, or 4-chamber view under the guidance of color Doppler flow mapping. All studies were digitally stored to simplify offline reviewing and measurements. At each time point, three optimal profiles of peak diastolic Doppler flow velocities were measured, and the results were averaged.

Heart rate reserve (step E) was calculated as the peak/rest HR from a 12-lead EKG (Ciampi Q, 2021).

All steps were performed by the same sonographer/cardiologist with the same transducer for cardiac, lung, and coronary scan although occasionally, a different high-frequency transducer was used for the coronary flow. All steps were acquired at rest and peak stress. If needed, steps were repeated after 5 min in the recovery phase.

A detailed visual description of the scanning procedure is also available in a 9-min movie from the consortium (YouTube. ABCDE SE 2030: How I do it. More easily done than said. Available at https://www.youtube.com/watch?v=O4-5FjSF7ao accessed September 26th, 2021).

### SE positivity criteria

All positivity criteria were determined a priori.

The A criterion was considered positive in presence of stress-induced regional wall motion abnormalities (WMSI stress > rest) when at least two adjacent segments of the same vascular territory of the left ventricle showed an increment of at least one point of the segmental score during SE.

The B criterion was considered positive in the presence of stress or rest B-lines ≥ 2 units (Ciampi Q, 2021).

The C criterion was considered positive in presence of force-based LVCR ≤ 1.1 for dipyridamole and ≤ 2.0 for dobutamine or exercise (Ciampi Q, 2021).

The D criterion was considered positive in presence of coronary flow velocity reserve ≤ 2.0 (Ciampi Q, 2021).

The E criterion was considered positive in presence of heart rate reserve < 1.22 for dipyridamole and ≤ 1.80 for dobutamine or exercise (Ciampi Q, 2021).

As required by SE 2020 protocol, all readers had passed the quality control for each of the 4 imaging parameters upstream to start patient recruitment (Ciampi Q, 2021).

SE response was summarized with a score ranging from 0 to 5 as follows: score 0 (all ABCDE markers within normal limits) or score 1–5, according to the number of abnormal steps (e. g. score 5 indicated all 5 steps were abnormal).

Inter- and intra-observer reproducibility was > 90% for all tested SE parameters as previously shown (Lang RM, 2015). Assessors were blinded to air quality data.

### Air quality data

Local air quality data were obtained from publicly available data sets from the regional authority of environmental protection. The air quality network managed by the environmental agency Arpa Campania consists of three stations measuring PM_2.5_, PM_10_, and NO_2,_ and 2 stations also measuring ozone. Although located in different sites, the stations show a high correlation ranging from 0.50 and 0.64 for NO_2_ and between 0.70 and 0.79 for PM_10_. For each patient and each test, the values of 2 particulate and 2 gaseous pollutants were collected (Environmental Protection Agency, 2014): PM_2.5_, PM_10_, NO_2_, and, which was available in a subset of 1,186 patients. Values of the same day of testing were taken as representative of that specific condition using the air monitor named CS which was about 2 km from the hospital where the cardiac functional test was carried out. The CS station presented hourly data for NO_2_ and ozone and daily data for PM_2.5_ and PM_10_. As a measure of exposure, the average concentration of NO_2_ and ozone in the hourly interval 8 p.m.–1 p.m. and the daily average of PM_2.5_, PM_10_ were considered. The following procedure for imputation of missing data was carried out. For the NO_2_ series, we considered an average of the values of the previous day and the following day if both were available. In the absence of one of the 2 variables, the value was estimated by considering the concentration data of the BN32 monitoring station at the same times scaled by the ratio of the annual average concentrations of the 2 monitoring stations. For the PM_2.5_PM_10_ series, it has been taken into account that the 2 concentrations are closely correlated. In case of the absence of one of the 2 variables, the missing data were estimated by the other one by taking into account the annual average ratio between PM_2.5_ and PM_10_. In the absence of both variables, the same imputation procedure was followed for NO_2_. Air quality data were collected and inputted by assessors (AR, CM) unaware of the patient identity, condition, and functional test findings. Values were collected from Centro Meteorologico e Climatologico (CEMEC, Meteorologic and Climatologic Center) of Arpa Campania https://www.arpacampania.it/web/guest/qualita-dell-aria.

### Statistical analysis

Categorical data are expressed in terms of the number of subjects and percentage while continuous data are expressed as mean ± standard deviation or median (minimum–maximum) depending on variables’ distribution. Linear regression analysis was used to assess the correlation between functional test results and air quality data. Independent predictors of B-lines were assessed by multivariable logistic regression analysis. Odds ratios (ORs) with the corresponding 95% confidence interval (CI) were estimated. A significance of 0.05 was required for a variable to be included in the multivariate model, while 0.1 was the cut-off value for exclusion. Statistical significance was set at *p* < 0.05. All analyses were performed using Statistical Package for the Social Sciences (IBM, SPSS Statistics, version 21).

## Results

The patients’ characteristics are reported in Table 1. One thousand one hundred eighty-eight (92%) patients had preserved (> 50%), 79 (6%) patients mid-range (20–49%) and 25 (2%) patients reduced (< 40%) resting ejection fraction (Table 1). Obstructive significant coronary artery disease was defined by a quantitatively assessed coronary diameter reduction ≥ 50% in the view showing the most severe stenosis and was present in 242 patients. One hundred ninety-seven (15%) patients had dyspnea as presenting symptom or chief complaint.

**Table 1:** Study population

Variable	Overall population (*N* = 1292)
Male/female sex, *n* (%)	857 (66%)/435 (34%)
Age, years	66 ± 10
BMI, kg/m^2^	28 ± 4
Known CAD/known HF	1095 (85%) / 197 (15%)
Previous PCI/CABG	664 (51%)
Hypertension, *n* (%)	1105 (85%)
Diabetes mellitus, *n* (%)	356 (28%)
Ejection fraction, %	60 ± 7
Beta-blockers, *n* (%)	840 (65%)
ACE-inhibitors or ARBs, *n* (%)	889 (69%)
Ca-antagonist, *n* (%)	109 (8%)
Diuretics, *n* (%)	245 (19%)

*ACE*, angiotensin-converting-enzyme; *ARB*, angiotensin II-receptor blockers; *BMI*, body mass index; *CABG*, coronary artery bypass grafting; *CAD*, coronary artery disease; *HF*, heart failure; *MR*, mitral regurgitation; *PCI*, percutaneous coronary intervention

### Rest and SE findings

Main rest TTE and SE findings are reported in Table 2; 183 patients (14%) showed B-lines at rest and 247 patients (19%) during stress.

**Table 2:** Main rest TTE and SE findings

	Values
A-step	
Rest WMSI	1.11 ± 0.26
Stress WMSI	1.11 ± 0.24
Δ-WMSI	− 0.00 ± 0.11
A-positivity, *n* (%)	59 (5%)
B-step	
Rest B-lines	0.7 [0–34]
Stress B-lines	1.1 [0–40]
B-positivity *n* (%)	247 (19%)
C-step	
Rest EF, %	60 ± 7
Rest force (mmHg/ml)	4.6 ± 1.6
Stress EF, %	74 ± 9
Stress force (mmHg/ml)	7.1 ± 3.3
Force	1.53 ± 0.42
C-force positivity, *n* (%)	202 (16%)
D-step	
Rest CFV, cm/s	25 ± 7
Stress CFV, cm/s	59 ± 19
CFVR	2.39 ± 0.37
D positivity, *n* (%)	167 (13%)
E-step	
Rest HR, bpm	66 ± 11
Peak HR, bpm	89 ± 15
HRR	1.36 ± 0.21
E-positivity, *n* (%)	387 (30%)

Values are expressed as mean ± standard deviation. CFV, coronary flow velocity; CFVR, coronary flow velocity reserve; EF, ejection fraction; HR, heart rate; HRR, heart rate reserve; LVCR, left ventricular contractile reserve; WMSI, wall motion score index

### Air quality findings

Air quality findings are reported in Table 3. All values were, on average, within legally allowed limits, in particular for NO_2_ (allowed limit: 40 µg/m^3^) and PM_2.5_ (allowed limit: 25 µg/m^3^).

**Table 3:** Air quality statistics in the same morning of testing

Pollutant	Mean concentration (µg/m^3^)	Standard deviation
NO_2_	21.4	10.1
Ozone	48.3	26.1
PM_2.5_	18.5	30.7
PM_10_	28.7	33.6

### Correlation between air quality and SE results

**Fig. 1:**
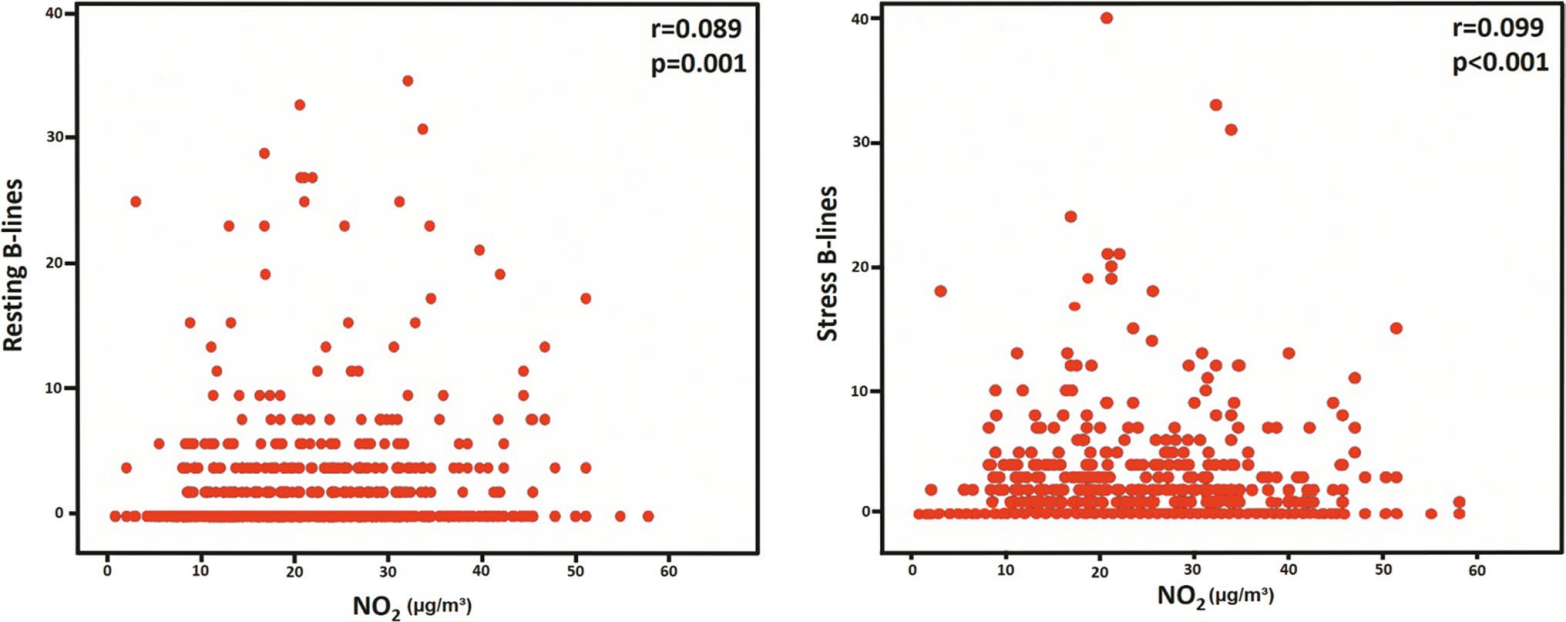
The correlation between percent NO_2_ and B-lines at rest (left panel) and during stress (right panel). x-axis: B-lines number; y-axis: NO_2_ values

For NO_2_, there was a significant but weak positive correlation with B-lines at rest (*r* = 0.089, *p* = 0.001) and during stress (*r* = 0.099, *p* < 0.001) (Fig. 1). There was an even weaker correlation of B-lines with PM_2.5_ and an inverse correlation with ozone concentrations (Table 4). All other air quality parameters did not show any significant correlation with other tested parameters of ischemia (step A), left ventricular contractile reserve (step C), coronary flow velocity reserve (step D), and heart rate reserve (step E), as detailed in Table 4. A multivariable logistic regression analysis, NO_2_ values above the median (23.1 µg/m^3^), independently predicted stress B-lines with odds ratio = 1.480 (95% CI 1.118–1.958) together with age, hypertension, diabetes, and reduced (< 50%) ejection fraction (Table 5).

**Table 4:** Correlation between air quality and ultrasound findings

	NO_2_	O_3_	PM_2.5_	PM_10_
Rest WMSI	*r* = 0.036 *p* = 0.193	*r* = 0.004 *p* = 0.886	*r* = − 0.018 *p* = 0.519	*r* = − 0.038 *p* = 0.176
Stress WMSI	*r* = 0.036 *p* = 0.196	*r* = − 0.021 *p* = 0.486	*r* = − 0.009 *p* = 0.741	*r* = − 0.025 *p* = 0.368
Rest B-lines	***r* = 0.089** *p* = 0.001**	***r* = ** − **0.066** *p* = 0.024*	*r* = 0.023 *p* = 0.416	*r* = 0.018 *p* = 0.525
Stress B-lines	***r* = 0.099** p < 0.001**	*r* = − 0.055 *p* = 0.061	*r* = 0.010 *p* = 0.715	*r* = 0.007 *p* = 0.805
Rest EF, %	*r* = − 0.012 *p* = 0.656	*r* = 0.003 *p* = 0.916	*r* = − 0.004 *p* = 0.898	*r* = 0.012 *p* = 0.666
Stress EF, %	*r* = 0.012 *p* = 0.679	*r* = 0.017 *p* = 0.573	*r* = − 0.012 *p* = 0.670	*r* = 0.018 *p* = 0.523
LVCR	*r* = − 0.016 *p* = 0.555	*r* = 0.034 *p* = 0.248	*r* = − 0.022 *p* = 0.430	*r* = − 0.022 *p* = 0.419
CFVR	*r* = − 0.005 *p* = 0.845	*r* = 0.037 *p* = 0.217	*r* = − 0.021 *p* = 0.448	*r* = − 0.027 *p* = 0.327
HRR	*r* = − 0.030 *p* = 0.288	*r* = 0.062 *p* = 0.037	*r* = − 0.036 *p* = 0.198	*r* = − 0.027 *p* = 0.328

Bold entries indicate statistical significance

^*^p < .05; **p < .01. Abbreviations as in Tables 2 and 3

**Table 5:** Predictors of peak stress B-lines with logistic regression analysis

	Univariable logistic regression analysis		Multivariable logistic regression analysis	
Variables	OR (95%CI)	*P*	OR (95%CI)	*P*
Age (years)	1.048 (1.032–1.063)	< *0.001*	1.045 (1.028–1.062)	< *0.001*
Sex (male)	2.233 (1.601–3.114)	< *0.001*	2.243 (1.579–3.184)	< *0.001*
Hypertension	1.201 (1.201–3.063)	*0.006*		
Diabetes	1.962 (1.466–2.626)	< *0.001*	1.811 (1.331–2.465)	< *0.001*
Prior MI	1.798 (1.355–2.384)	< *0.001*	1.393 (1.026–1.892)	*0.033*
Reduced EF (< 50%)	3.460 (2.274–5.265)	< *0.001*	2.734 (1.741–4.294)	< *0.001*
NO2 > median*	1.480 (1.118–1.958)	*0.006*	1.464 (1.090–1.968)	*0.011*

^*^Above 23.1 µg/m^3^

## Discussion

**Fig. 2:**
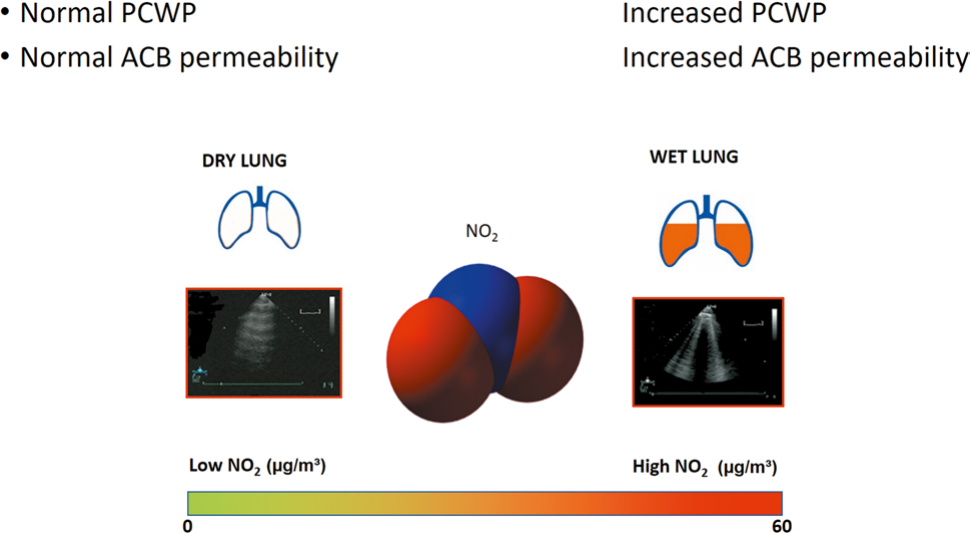
An increase of NO_2_ in ambient air increases the vulnerability of the lung to develop pulmonary congestion at rest and during stress, possibly acting on increased permeability of the alveolar-capillary barrier (ACB) for any given increase in pulmonary capillary wedge pressure (PCWP)

Air pollution may affect the results of cardiac functional testing, although not all the components of air pollution have the same impact and not all aspects of cardiac functional testing show the same vulnerability to air pollution components. In particular, we found that the increase in NO_2_ is especially toxic for vulnerability to lung congestion mirrored by B-lines in clinically stable patients (Fig. 2). NO_2_ may exert a detrimental cardiovascular effect through augmented inflammatory and oxyradical stress at the lung, heart, and systemic levels (Brook RD, 2010). The increased production of inflammatory cytokines by leukocytes alters the alveolar-capillary barrier increasing its permeability to water filtration into the lung extravascular space for any given intravascular pressure (Chiu PF, 2019). Among the components of air pollution, NO_2_ is likely to be the most toxic for the alveolar-capillary barrier, which is the entry point of NO_2_ and also the key factor in the transition from stable heart failure to acute decompensated heart failure requiring hospital admission (Pappas and Filippatos 2011). Other pollutants (such as PM_2.5_) may chronically contribute more strongly to the development of heart failure in the long-term independently and incrementally over NO_2_, but NO_2_ may exert a prominent toxic effect on the alveolar-capillary membrane. The extreme demonstration of the direct toxic effects of NO_2_ is the symptoms of pulmonary edema acutely found in healthy subjects exposed to NO_2_ concentrations 10- or 100-times higher than allowed limits, as it happens for instance in silos filler’s disease, numismatist’s pneumonia, explosive detonation, fire workers’ poisoning, or ice hockey lung (Nash T, 1990). In all these conditions, the poorly soluble NO_2_ gas, heavier than air, penetrates the peripheral airways and alveoli and generates toxic nitrous and nitric acid after combining with water, thereby increasing cell membrane permeability resulting in interstitial pulmonary edema (Brat K, 2013), easily detectable as B-lines. In the experimental animal, the increase in inhaled NO_2_ concentration produces linear exposure-related lung edema (Vassilyadi M, 1988). NO_2_ concentration is substantially lower in our study setting but enrolled patients are on average more susceptible, i.e. at higher risk for cardiovascular events than a general population for a given level of pollution exposure.

### Comparison with previous studies

In patients with coronary artery disease, the increase in NO_2_ in the days or hours before testing was associated with greater signs of pulmonary congestion during exercise or pharmacological stress in 19 patients studied before and after lockdown, when substantial air cleaning occurred for sudden traffic ban and industry restrictions (D’Andrea A, 2021). The short-term increase in NO_2_ is also associated with a same-day increase in admissions for acute heart failure, which is a life-threatening event mostly characterized by pulmonary congestion and distress of the alveolar-capillary barrier (Wellenius GA, 2005). In a 2013 meta-analysis on 35 articles, air pollution, and in particular, the increase in NO_2_, PM_2.5_, and carbon monoxide showed a close temporal association with same-day heart failure hospitalization and death (Shah AS, 2013). In a study on 26 large Chinese cities conducted between 2014 and 2016, an interquartile range increase in nitrogen dioxide corresponded to a 1.6% increase on the current day hospital admissions for heart failure (Liu H, 2018). NO_2_ concentration is also significantly associated with the development of incident heart failure with a risk of 1.10 for every 10 μg/m^3^ increase in concentration, in a study on 432,539 participants initially free of heart failure and followed-up for a median of 10.1 years (Wang M, 2021a). In a multilocation analysis in 398 cities, there was an independent and linear association between short-term (same day or day before) exposure to NO_2_ and total, cardiovascular and respiratory mortality, with a linear dose–response curve without discernible thresholds (Meng X, 2021).

We found a weak protective effect of ozone on the development of B-lines. This can appear paradoxical since ozone is a recognized risk factor for cardiovascular events, independent and additive over PM_2.5_, and in its turn has complex cardiovascular detrimental effects, including stimulation of sympathetic activity and arterial vasoconstriction. However, ozone at ground level is a secondary pollutant destroyed by NO_2_, so that ozone levels can fall when NO_2_ concentration decreases (Schipa I, 2009).

### Clinical implications

Risk stratification and phenotyping of disease are based upon response to specific variables and at least some of them such as B-lines can be affected by changes in air pollution falling well within the range of acceptable values, set at 40 mcg/m^3^ (annual average) by the European Union and World Health Organization (Al-Kindi SG, 2020). Our findings suggest that even normal or tolerable NO_2_ concentrations can have detectable adverse effects on pulmonary congestion detected by lung ultrasound. The recognition of this variable is especially important since air pollution can be considered today an actionable therapeutic target, for instance with air cleaners and personal protection devices such as face masks (Rajagopalan S, 2020).

### Study limitations

The study is observational, with all potential confounders of a non-randomized design. The association between B-lines and NO_2_ concentration in ambient air was present but weak, and further studies an environment with high pollution levels are needed to corroborate these findings obtained with tolerable levels of air pollution, although linear non-threshold models are usually considered adequate to explain the effects of NO_2_ (Samoli E, 2003).

Air quality cannot be characterized by a single parameter, and each component of the complex mixture of particles, gases, and liquids contributing to air quality can have independent effects (Cao R, 2021). Exogenous exposures should be simultaneously combined with the assessment of endogenous exposures and modifiable risk factors to have a more comprehensive assessment of the exposome (Tang S, 2021). The air pollution assessment with monitoring close to the clinical department cannot reflect the actual individual exposure, but it is an acceptable proxy when no wearable air pollutants monitoring is possible.

We used the same-day exposure, and we did not assess the previous 30 days, or day 1 before testing, or the year before testing. The exposure was assessed in the 6 h on the testing morning. Air pollutants can induce lung injury via endothelial inflammation and dysfunction, and acute exposure to nitrogen dioxide is associated with the elevation of proinflammatory circulating factors (Channel MM, 2012).

Long-term exposure (e.g. annual mean or multiple-year averages) surely matters in determining all-cause, cardiovascular and respiratory mortality from exposure to nitrogen dioxide (Huang S, 2021). However, the same-day exposure on the morning of testing is more likely to reflect the condition at the time of testing and has shown a powerful relationship with same-day mortality (Wang M, 2021b) or same-day admissions for heart failure (Lee DW 2021).

However, the same-day exposure on the morning of testing is more likely to reflect the condition at the time of testing and has shown a powerful relationship with same-day mortality or same-day admissions for heart failure (Lee DW, 2021).

### Conclusion

An increase in NO_2_ well within the European Union air quality standards is associated with worsening pulmonary congestion at rest and during stress. Changes in air quality did not affect imaging biomarkers of inducible ischemia, contractile reserve, coronary flow velocity reserve, or cardiac autonomic function. Local air quality data are easily available in real-time with freely downloadable apps on the smartphone and individual exposures can be monitored with wearable pollution detectors. NO_2_ levels in ambient air should probably be considered in the evaluation of pulmonary congestion assessed with B-lines by lung ultrasound.

## Data Availability

Supporting data set will be made available upon reasonable request.

## Abbreviations

NO_2_: Nitrogen dioxide
PM_2.5_: Fine particulate matter with aerodynamic diameter < 2.5 µm
PM_10_: Particulate matter with aerodynamic diameter < 10 µm
SE: Stress echocardiography
TTE: Transthoracic echocardiography
WMSI: Wall motion score index
